# Chronic pain and premature mortality in men and women, using data from UK Biobank. Reply.

**DOI:** 10.1172/JCI168070

**Published:** 2023-03-01

**Authors:** Marc Parisien, Audrey V. Grant, Arjun Muralidharan, Luda Diatchenko, Jeffrey S. Mogil

**Affiliations:** 1 Alan Edwards Centre for Research on Pain, McGill University, Montreal, Quebec, Canada.; 2 Charles Perkin Centre, Faculty of Science, University of Sydney, New South Wales, Australia.

**Keywords:** Neuroscience, Pain

## The authors reply:

In a Letter to the Editor regarding our recently published paper (1), Macfarlane and colleagues (2) argue that our method of analysis of data from the UK Biobank (UKB) project was inappropriate and that we came to an incorrect conclusion regarding a potential sex-dependent effect of pain on mortality. We thank these excellent scientists for engaging with our work.

Our original analysis asked if there was a relationship between age at death and pain severity. We were particularly interested in pain severity as an independent measure, as opposed to simple presence of chronic widespread pain (CWP), because of the results of our recent paper (3) — which, although not yet published, were known to us at the time — showing a correlation between pain severity and lifespan in male but not female mice. Pain severity data have only been made available recently in the UKB, and these data only reflect a subset of participants. The closest available proxy for pain severity in the UKB was established to be the number of chronic pain sites; indeed, both pain phenotypes are strongly correlated (4).

**Figure 1:**
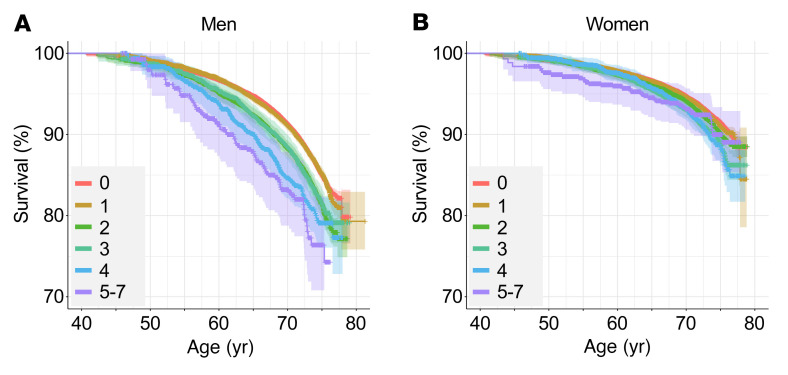
Survival trajectories in the UK Biobank. Group survival tracked as a function of (**A**) male and (**B**) female participant age in years. Trajectories stratified by the number of chronic pain sites. Individuals with 5–7 chronic pain sites were grouped together to increase sample size. Shaded areas highlight CIs.

With the critique by Macfarlane et al. in mind, we have added to our previous analysis. We identified persons who reported chronic pain at the time of recruitment between 2006 and 2010 and prospectively identified deaths up until February 2016, in the same spirit as what was done by Macfarlane’s group previously (5). Each participant was assigned a number of endorsed chronic pain sites from 0 to 7. Survival plots that by definition included those who were alive at baseline were made with the “ggsurvplot” function of the “survminer” R package, using age as the time scale. In the present scenario, we expected the hazard to change as a function of age rather than time under observation, given the wide range of baseline ages. Our data were left truncated and right censored: the analysis was based on UKB’s census time period for each participant, from their age at recruitment until their age either at death or in February 2016. The optimum start time would be age at chronic pain status onset, but this was unknown. We observed a visually striking difference between men (Figure 1A) and women (Figure 1B), even taking into account the known higher mortality rate in men than women.

We attempted to answer whether there was a significant sex difference in the relationship between the number of chronic pain sites and mortality by using a Cox regression. An age × phenotype (i.e., number of pain sites) term was added to the regression in addition to age to account for the strong association between age and number of chronic pain sites at recruitment (as opposed to age as a time scale), bettering the overall fit of the Cox model. The regression results indicated that each additional chronic pain site increased risk for death (with a HR = 1.7) and that BMI and alcohol consumption were additional risk factors (HR > 1), as already substantiated in the literature. The analysis also showed that men died at a greater rate than women (HR = 1.9), a positive control. Despite all this, we found a sex × phenotype interaction of P = 0.05, with a HR = 1.03 (> 1), indicating that within a given number of chronic pain sites, men had additional mortality risk. Schoenfeld’s test for the sex × phenotype interaction was not significant (*P* = 0.82), indicative of no departure from time independence.

The bigger question of whether there is indeed a sex-specific relationship between chronic pain and longevity in humans, as there clearly is in mice (1, 3), remains open; a more definitive answer will be obtainable once survival percentages descend to approximately 50%. We would remind the reader, however, that the main focus of our paper (1) was the sex-dependent role of telomere dysfunction and cellular senescence on chronic pain (e.g., the male-specific *TP53* gene association), not sex‑dependent pain epidemiology per se.

## Version 1 03/01/2023

Electronic publication
